# Supplementary material for: Genes and pathways determining flowering time variation in temperate‐adapted sorghum
Source: Plant J. 2025 Jun 2;122(5):e70250. doi: 10.1111/tpj.70250 (PMC12129571; doi:10.1111/tpj.70250)
Supplement: Supplementary file 1 — Figure S1. Distributions and correlations flowering time within and between Nebraska and Alabama. Figure S2. Relationship between flowering time variation and leaf number. Figure S3. Distribution of pseudoalignment rates for each RNA‐seq sample used in this study. Figure S4. Sorghum around the world and PCs with relation to the sub‐population of sorghum. Figure S5. Results of a genome wide association study conducted using only lines from the Sorghum Association Panel. Figure S6. Linkage disequilibrium for the most significant SNPs found in the GWAS analysis. Figure S7. Correlation between the expression of sbi‐MIR156h and the three SBP transcription factors identified via TWAS. Figure S8. Position of mir172a relative to the flowering time associated marker Chr09:62,620,720. Figure S9. Phylogenetic tree for FT genes Phyogenetic relationships among the set of FT‐like genes found in the sorghum, maize, rice, and arabidopsis genomes. Table S1. Annotated sorghum gene models and functional annotations located within 100 kilobases of the flowering time associated genetic marker located at Chr09:62,620,720. Table S2. Names and gene model identifiers for the thirteen FT‐like sorghum genes described by (Wolabu et al., 2016), as well as names and gene model identifiers for syntenic orthologs of these sorghum genes in the genomes of rice, and both maize subgenomes. Table S3. Average expression level and significance of association with flowering time for all annotated FT‐like genes in the sorghum genome. Table S4. Genes located within the plausible window, defined based on linage disequilibrium, to represent potential causal variations associated with the three most significant SNPs identified in the GWAS analysis for flowering time variation. [file TPJ-122-0-s001.pdf]

Additional Information

Supplementary Figures

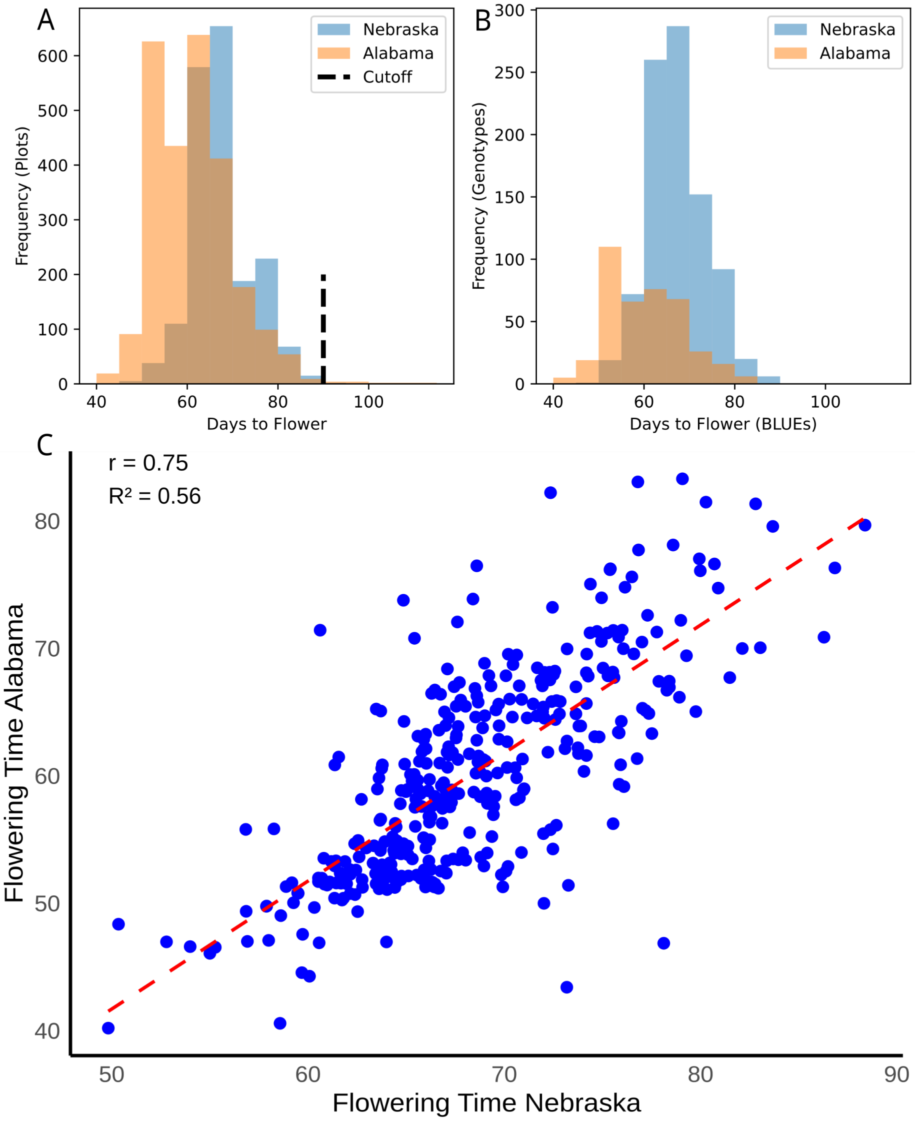

Figure S1. Distributions and correlations flowering time within and between Nebraska and Alabama. A) The distribution raw, plot level values for flowering time (i.e. not spatially corrected and not aggregated across multiple observations of the same genotype) in each environment. Vertical dashed black line indicates the cutoff used to exclude extremely late flowering plots B) The distribution of spatially corrected BLUEs for each genotype in each environment. C) The relationship between spatially corrected best unbiased linear estimators in the Alabama (y-axis) and Nebraska (x-axis) field experiments. A dashed red line indicates the best fit linear regression. N = 365 (for the genotypes shared between the Nebraska and Alabama)

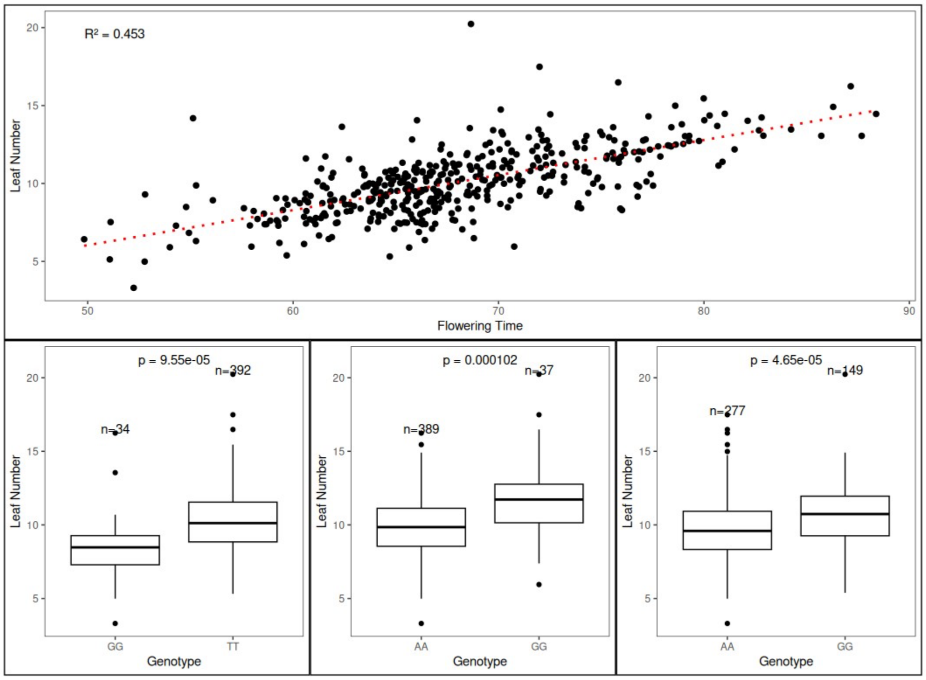

Figure S2. Relationship between flowering time variation and leaf number. A) Correlation between flowering time and extant leaf number for a subset of 427 sorghum genotypes for which RNA-seq derived genetic markers were available, flowering time was scored, and leaf number was recorded. B) Difference in leaf number between sorghum genotypes homozygous for either the T or G allele at Chr03:69,067,236, a genetic marker associated with significant variation in flowering time in this population. p-value shown calculated using a two-tailed t-test. C) Difference in leaf number between sorghum genotypes homozygous for either the A or G allele at Chr06:39,694,475, a genetic marker associated with significant variation in flowering time in this population. D) Difference in leaf number between sorghum genotypes homozygous for either the A or G allele at Chr06:39,694,475, a genetic marker associated with significant variation in flowering time in this population. One sorghum variety with a heterozygous genotype call for this marker was excluded.

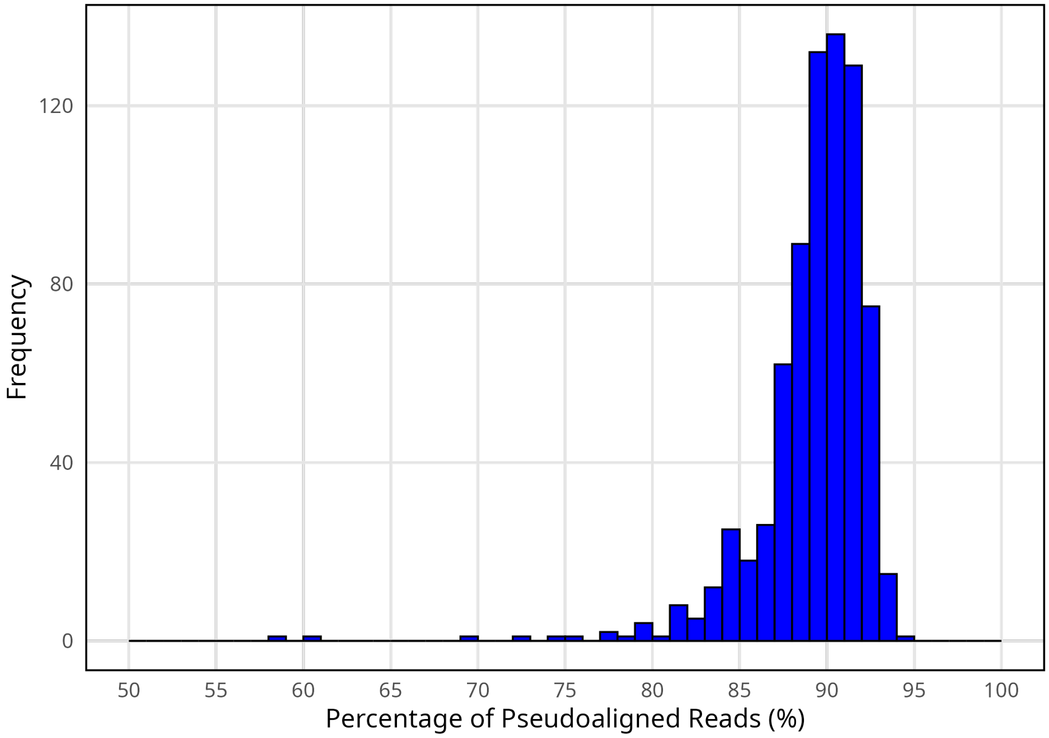

Figure S3. Distribution of pseudoalignment rates for each RNA-seq sample used in this study. Proportion of reads that could be pseudoaligned to the "primary transcript only" transcript sequences of the BTx623 v5 sorghum reference genome via kallisto. One extremely low alignment sample, PI 656042, where only 8.9% of reads could be matched to sorghum transcripts, is omitted from this visualization to aid in readability.

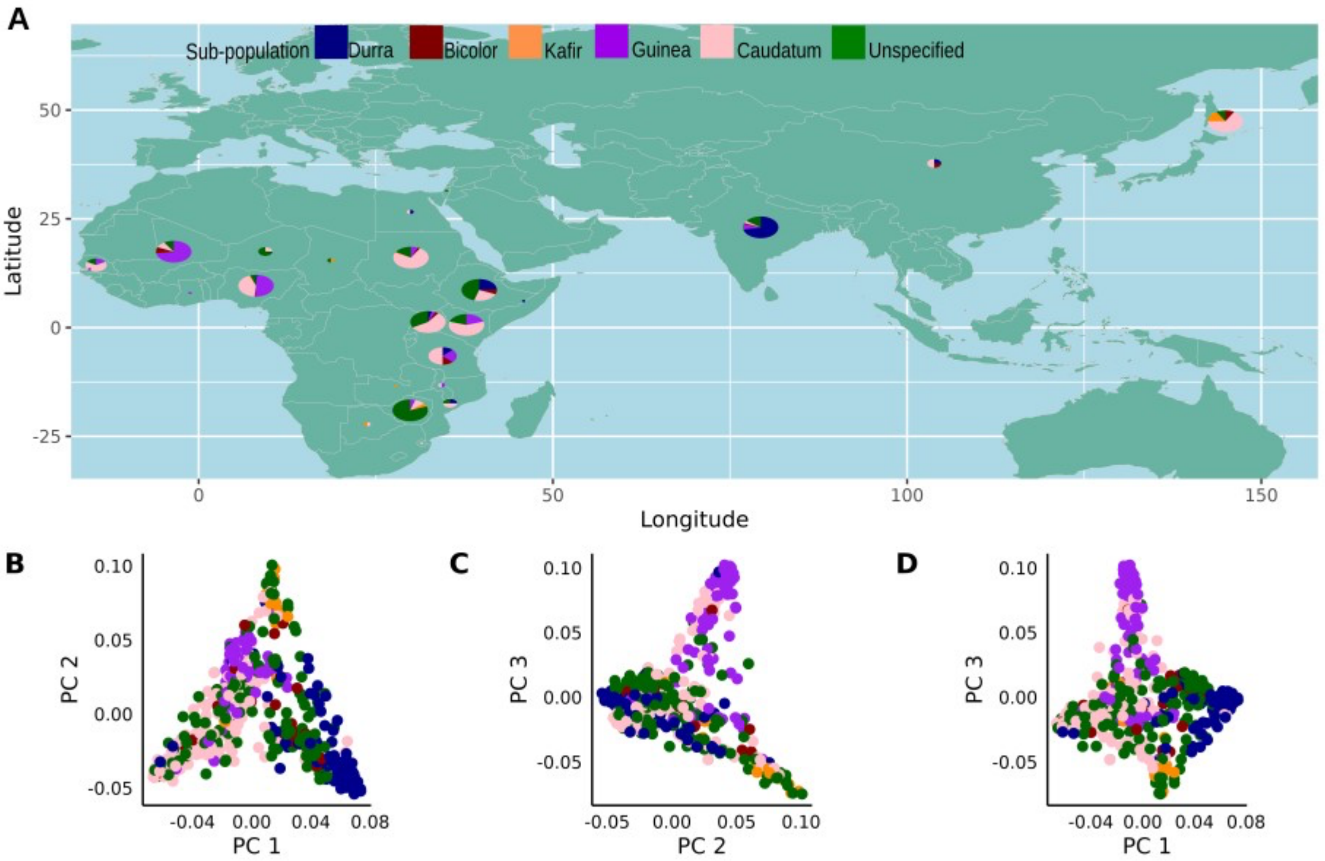

Figure S4. Sorghum around the world and PCs with relation to the sub-population of sorghum: A. World Map for the Sorghum Diversity Panel and the places around the world with respect to the sub-population. The Principal Components are generated using SNP dataset B. Principal Component 1 with respect to Principal Component 2 with the colors defining sub-population. C. Principal Component 2 with respect to Principal Component 3 with the colors defining sub-population. D. Principal Component 3 with respect to Principal Component 1 with the colors defining sub-population.

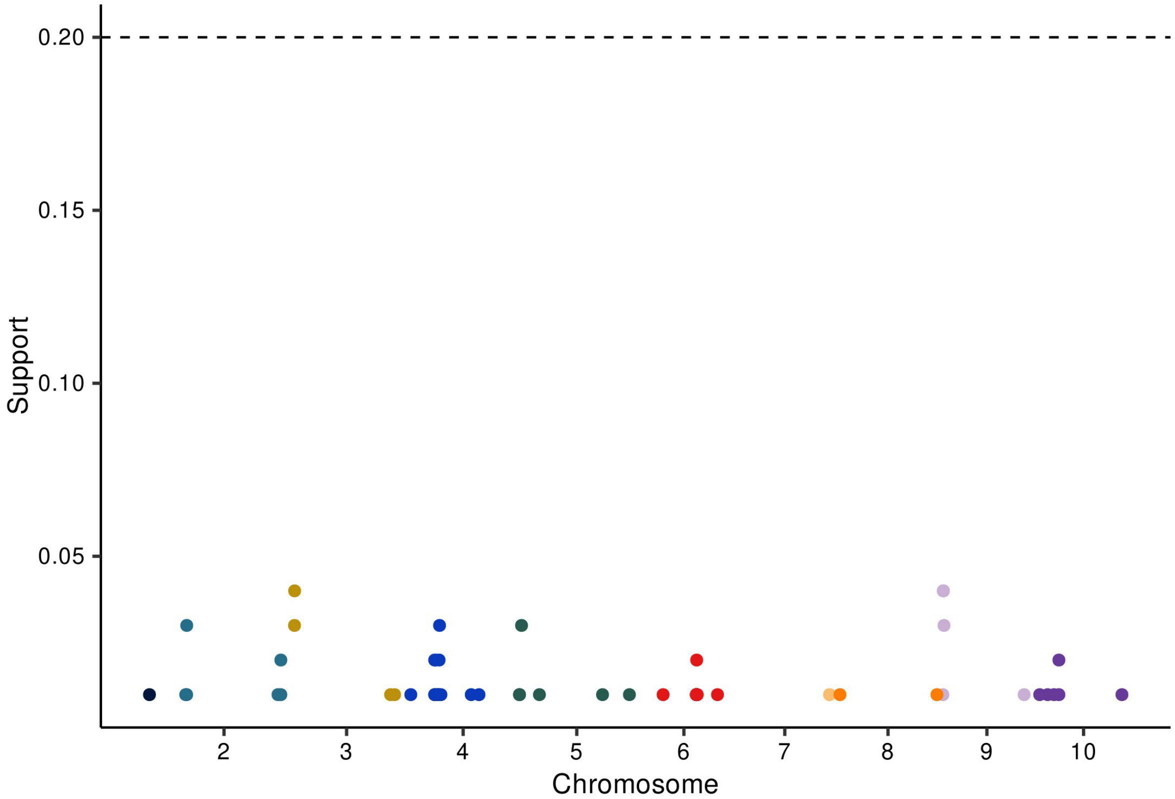

Figure S5. Results of a genome wide association study conducted using only lines from the Sorghum Association Panel. Genome wide association study conducted for flowering time measured across 303 sorghum genotypes scored in Lincoln, Nebraska in 2021 which are also part of the Sorghum Association Panel. Y-axis indicates the resampling model inclusion probability calculated from 100 iterations of the FarmCPU GWAS algorithm. Horizontal dashed line indicates the threshold employed to consider a marker significantly associated with flowering time in this study (RMIP = 0.2).

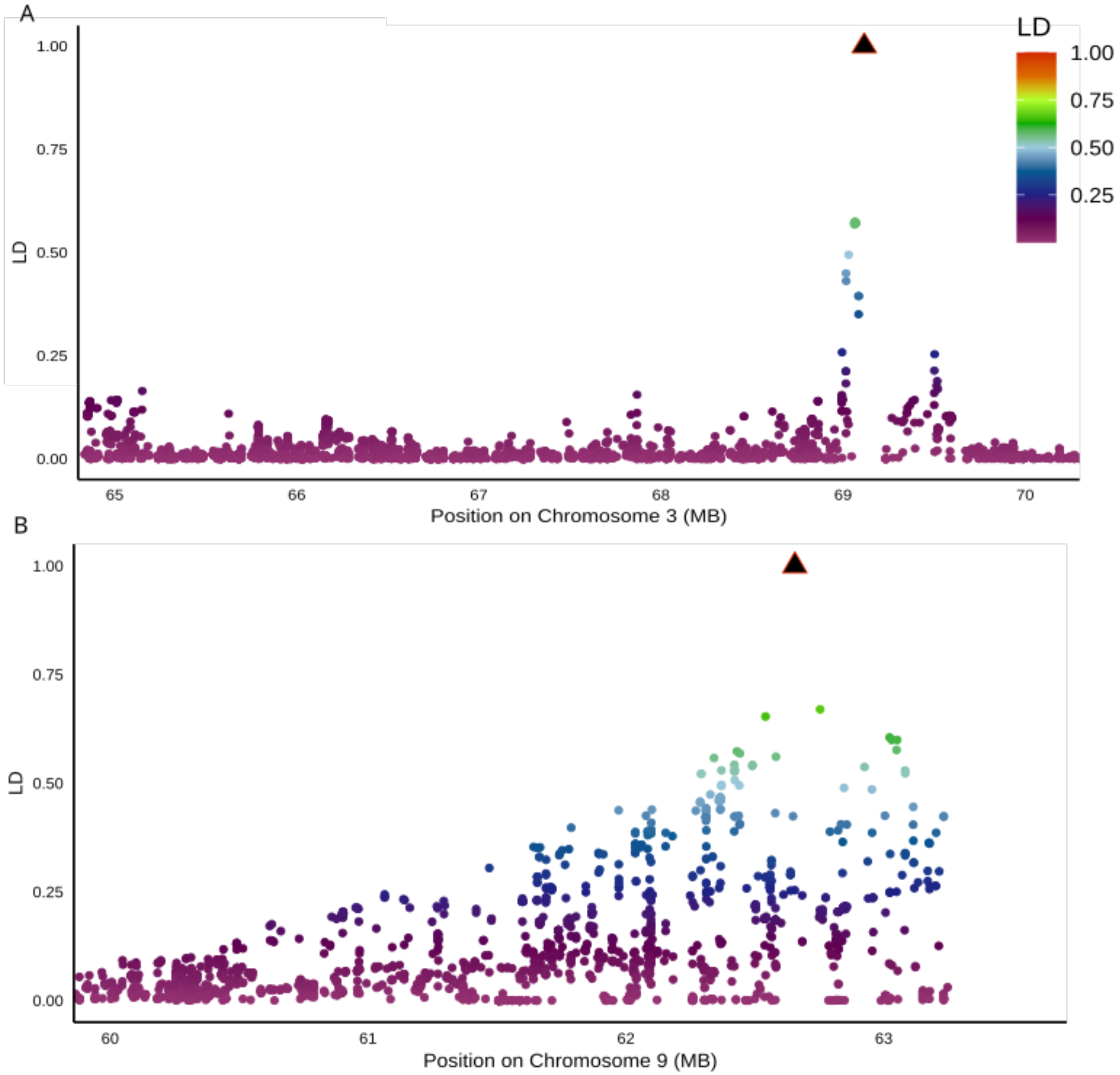

Figure S6. Linkage disequilibrium for the most significant SNPs found in the GWAS analysis. A) Genomic interval and annotated genes surrounding the trait associated marker Chr03:69,067,236. Each genetic marker within the interval is indicated with a circle whose color and position on the y-axis correspond to the degree of linkage disequilibrium between that marker and the trait associated marker. The black triangle shows the position of the trait associated marker. B) Genomic interval and annotated genes surrounding the trait associated marker Chr09:62,620,720.

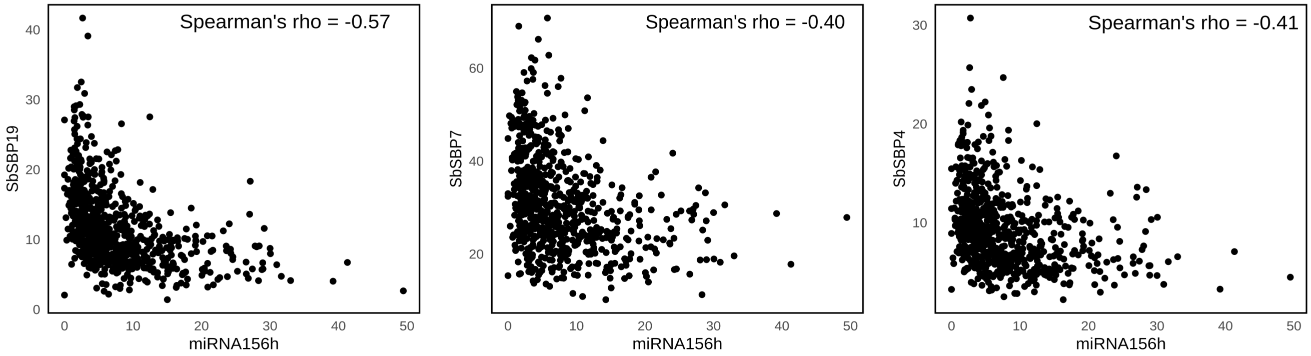

Figure S7. Correlation between the expression of sbi-MIR156h and the three SBP transcription factors identified via TWAS. Each point indicates the expression of sbi-MIR156h in TPM (x-axis) and the expression of the SBP transcription factor name given on the y-axis in units of TPM in one of the 738 sorghum RNA-seq samples analyzed as part of this study.

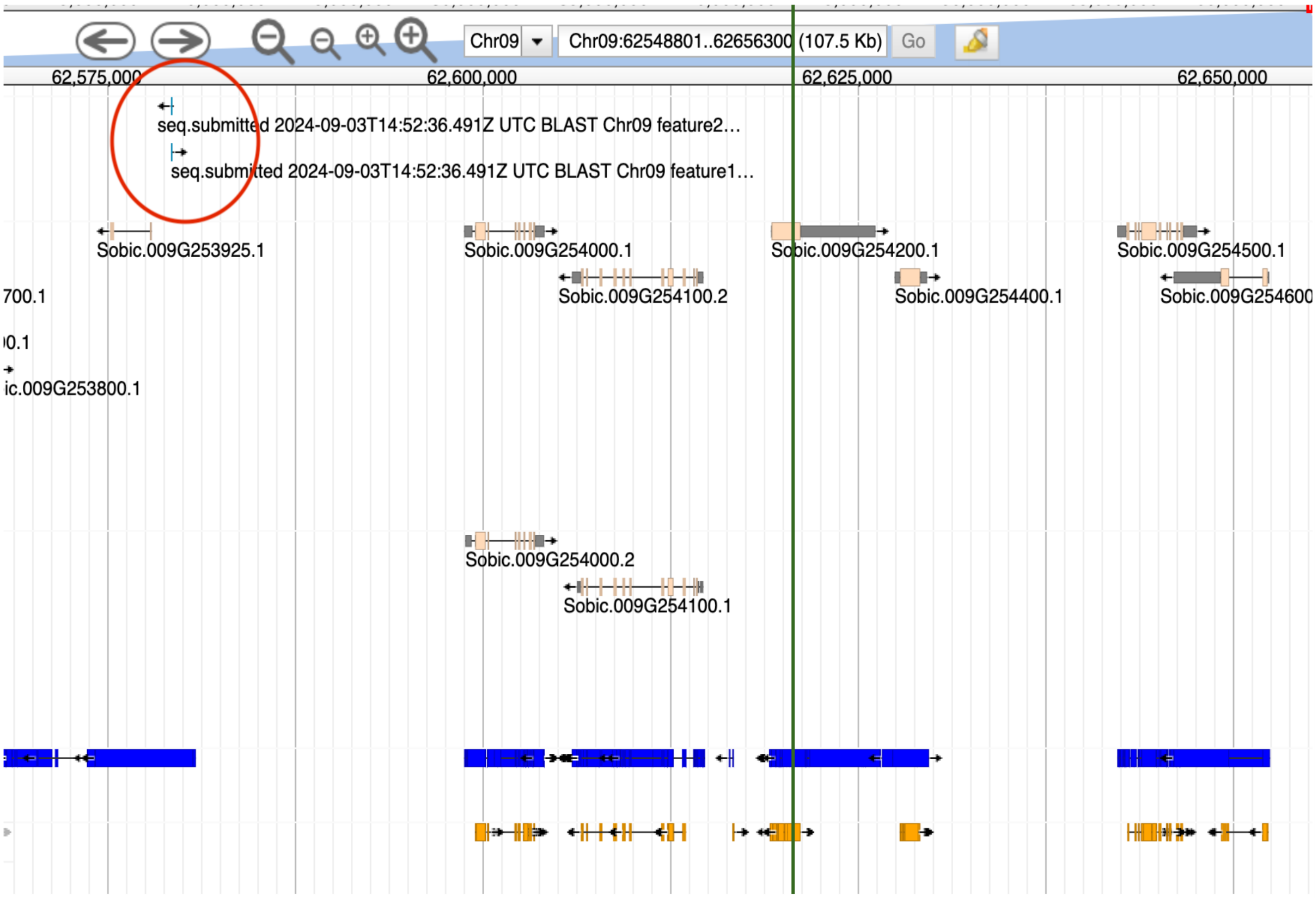

Figure S8. Position of mir172a relative to the flowering time associated marker Chr09:62,620,720. Genomic interval and annotated genes surrounding the trait associated marker Chr09:62,620,720. Screenshot from the phytozome genome browser showing the position of mir172a (red circle) and the position of Chr09:62,620,72 green verticle line.

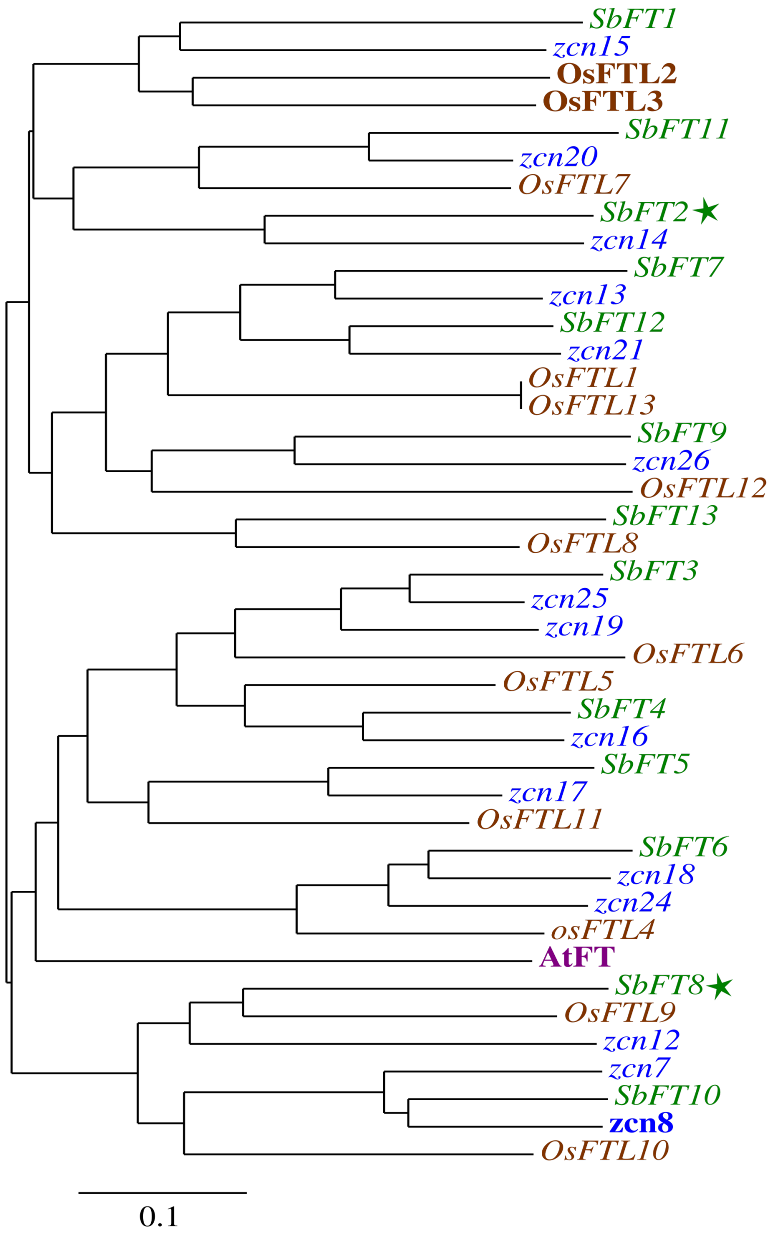

Figure S9. Phylogenetic tree for FT genes Phyogenetic relationships among the set of FT-like genes found in the sorghum, maize, rice, and arabidopsis genomes. Star indicates the sorghum gene copy linked to variation in flowering time via TWAS and GWAS in our study. Phylogeny constructed using PhyML using a multiple sequence generated by aligning the nucleotide sequence of the primary transcript for each gene with MUSCLE. Bold names indicate the direct promoter of flowering-time in the specific

Supplementary Tables

GeneID	Function
Sobic.009G253000	RNA polymerase sigma factor
Sobic.009G253101	Pentatricopeptide repeat containing protein

Sobic.009G253150	Pentatricopeptide repeat containing protein
Sobic.009G253200	RNA polymerase sigma factor
Sobic.009G253300	MYB transcription factor
Sobic.009G253400	Hyaluronan (RNA binding)
Sobic.009G253450	Unknown protein, no homologs
Sobic.009G253500	F-box domain containing protein
Sobic.009G253600	Pentatricopeptide repeat containing protein
Sobic.009G253700	Phosphatidylinositol 4-kinase
Sobic.009G253800	Aspartyl protease
Sobic.009G253925	Unknown protein, no homologs
Sobic.009G254000	Protein kinase
Sobic.009G254100	RNA helicase
Sobic.009G254200	Protein kinase
Sobic.009G254400	Leucine rich repeat protein.
Sobic.009G254500	Protein kinase
Sobic.009G254600	Alpha/beta hydrolase
Sobic.009G254700	Alpha/beta hydrolase fold-containing protein
Sobic.009G254750	Unknown protein, no homologs
Sobic.009G254800	Heat shock protein
Sobic.009G254900	Lysine decarboxylase
Sobic.009G255000	Protein kinase
Sobic.009G255100	Membrane protein homologous to hypersensitive response induced gene

Supplemental Table S1. Annotated sorghum gene models and functional annotations located within 100 kilobases of the flowering time associated genetic marker located at Chr09:62,620,720

Sorghum (v5)	Rice	Maize1	Maize2	Sorghum (v1)
SbFT1<br>Sobic.010G045100	osFTL2/Hd3a<br>LOC_Os06g06320<br>osFTL3<br>LOC_Os06g06300	NA	zcn15<br>Zm00001eb271180	Sb10g003940
SbFT2<br>Sobic.003G017200	osFTL1<br>LOC_Os01g11940	NA	zcn14<br>Zm00001eb338650	Sb03g001700
SbFT3<br>Sobic.006G128500	osFTL6<br>LOC_Os04g41130	zcn25<br>Zm00001eb078980	zcn19<br>Zm00001eb425430	Sb06g020850
SbFT4<br>Sobic.004G206600	osFTL5<br>LOC_Os02g39064	zcn16<br>Zm00001eb246470	NA	Sb04g025210

SbFT5 Sobic.005G110406	osFTL11 LOC_Os11g18870	zcn17 Zm00001eb090320	NA	Sb0010s003120
SbFT6 Sobic.002G262500	osFTL4 LOC_Os09g33850	zcn24 Zm00001eb318290	zcn18 Zm00001eb102650	Sb02g029725
SbFT7 Sobic.004G101800	osFTL13 LOC_Os02g13830	zcn13 Zm00001eb239360	NA	Sb04g008320
SbFT8 Sobic.003G295300	osFTL9 LOC_Os01g54490	zcn12 Zm00001eb153190	NA	Sb03g034580
SbFT9 Sobic.010G164200	osFTL12 LOC_Os06g35940	zcn26 Zm00001eb384770	NA	Sb10g021790
SbFT10 Sobic.009G199900	osFTL10 LOC_Os05g44180	zcn7 Zm00001eb293080	zcn8 Zm00001eb353250	Sb09g025760
SbFT11 Sobic.008G082200	osFTL7 LOC_Os12g13030	zcn20 Zm00001eb411580	NA	Sb08g008180
SbFT12 Sobic.006G047700	osFTL13 LOC_Os02g13830	zcn21 Zm00001eb085740	NA	Sb06g012260
SbFT13 Sobic.003G026600	osFTL8 LOC_Os01g10590	NA	NA	Sb03g002500

Supplemental Table S2. Names and gene model identifiers for the thirteen FT-like sorghum genes described by (Wolabu et al., 2016), as well as names and gene model identifiers for syntenic orthologs of these sorghum genes in the genomes of rice, and both maize subgenomes.

Gene Name	Gene Model	Mean Expression	p-value
SbFT1	Sobic.010G045100	49.43 TPM	0.0055
SbFT2	Sobic.003G017200	19.07 TPM	3.11E-06
SbFT3	Sobic.006G128500	3.01 TPM	0.097
SbFT4	Sobic.004G206600	0.78 TPM	0.25
SbFT5	Sobic.005G110406	0.00017 TPM	N/A
SbFT6	Sobic.002G262500	0.10 TPM	0.9
SbFT7	Sobic.004G101800	0.021 TPM	0.22
SbFT8	Sobic.003G295300	160.27 TPM	0.51
SbFT9	Sobic.010G164200	73.76 TPM	0.69
SbFT10	Sobic.009G199900	162.55 TPM	0.082
SbFT11	Sobic.008G082200	0.0025 TPM	N/A
SbFT12	Sobic.006G047700	0	N/A
SbFT13	Sobic.003G026600	8.44 TPM	0.98

Supplemental Table S3. Average expression level and significance of association with flowering time for all annotated FT-like genes in the sorghum genome

Gene	Chromosome	Start	End
Sobic.003G294800	3	69031106	69032301
Sobic.003G294900	3	69035561	69037443
Sobic.003G295000	3	69038797	69039496
Sobic.003G295100	3	69052628	69054680

Sobic.003G295200	3	69054905	69059485
Sobic.003G295300	3	69067022	69069004
Sobic.003G295400	3	69086977	69093407
Sobic.003G295500	3	69099061	69103679
Sobic.003G295600	3	69105009	69106441
Sobic.003G295700	3	69106746	69109406
Sobic.003G295800	3	69110057	69112616
Sobic.003G295900	3	69114588	69117290
Sobic.006G051300	6	39456582	39457338
Sobic.006G051700	6	39497695	39503452
Sobic.006G051800	6	39516620	39525297
Sobic.006G051900	6	39529707	39537269
Sobic.006G052001	6	39684643	39684987
Sobic.006G052100	6	39694491	39698895
Sobic.006G052200	6	39735882	39742705
Sobic.006G052300	6	39744446	39747698
Sobic.006G052400	6	39748691	39758701
Sobic.006G052500	6	39764940	39771839
Sobic.006G052650	6	39772213	39773607
Sobic.006G052800	6	39859535	39870930
Sobic.009G248600	9	62212543	62215125
Sobic.009G248700	9	62219851	62221401
Sobic.009G248802	9	62224278	62225147
Sobic.009G248900	9	62229293	62232477
Sobic.009G249000	9	62232406	62237451
Sobic.009G249100	9	62236066	62242580
Sobic.009G249200	9	62253093	62258489
Sobic.009G249400	9	62263243	62265854
Sobic.009G249500	9	62266072	62271442
Sobic.009G249600	9	62274496	62277437
Sobic.009G249700	9	62290262	62296360
Sobic.009G249800	9	62301245	62309636
Sobic.009G249900	9	62325155	62331431
Sobic.009G250000	9	62331747	62337155
Sobic.009G250100	9	62343576	62348750
Sobic.009G250200	9	62349111	62353243
Sobic.009G250300	9	62355175	62357219
Sobic.009G250400	9	62373942	62377839
Sobic.009G250500	9	62377854	62378941
Sobic.009G250600	9	62379821	62380603
Sobic.009G250650	9	62380604	62381297
Sobic.009G250700	9	62383850	62386826
Sobic.009G250800	9	62386827	62389039
Sobic.009G250900	9	62391709	62397674

Sobic.009G251000	9	62399542	62405414
Sobic.009G251100	9	62402346	62411399
Sobic.009G251200	9	62428208	62430745
Sobic.009G251300	9	62431311	62434200
Sobic.009G251400	9	62442909	62446141
Sobic.009G251500	9	62453010	62464397
Sobic.009G251600	9	62465473	62468108
Sobic.009G251700	9	62468311	62471140
Sobic.009G251800	9	62473289	62478526
Sobic.009G251901	9	62479704	62480222
Sobic.009G252000	9	62481154	62482957
Sobic.009G252100	9	62486869	62489573
Sobic.009G252200	9	62491462	62497155
Sobic.009G252300	9	62500102	62504783
Sobic.009G252400	9	62505516	62507904
Sobic.009G252500	9	62510531	62512728
Sobic.009G252600	9	62514058	62516159
Sobic.009G252700	9	62509233	62517391
Sobic.009G252800	9	62519995	62526011
Sobic.009G253000	9	62528831	62531756
Sobic.009G253101	9	62532501	62533289
Sobic.009G253150	9	62533371	62536916
Sobic.009G253200	9	62538152	62539125
Sobic.009G253300	9	62541917	62543252
Sobic.009G253400	9	62544429	62547953
Sobic.009G253450	9	62547354	62553419
Sobic.009G253500	9	62556761	62558056
Sobic.009G253600	9	62559552	62560529
Sobic.009G253700	9	62560627	62563111
Sobic.009G253800	9	62566025	62567884
Sobic.009G253925	9	62575197	62577971
Sobic.009G254000	9	62598787	62604134
Sobic.009G254100	9	62605966	62614719
Sobic.009G254200	9	62619237	62626188
Sobic.009G254400	9	62627475	62629626
Sobic.009G254500	9	62642299	62647612
Sobic.009G254600	9	62646059	62652375
Sobic.009G254700	9	62673645	62677357
Sobic.009G254750	9	62674178	62676662
Sobic.009G254800	9	62684256	62686062
Sobic.009G254900	9	62699952	62704490
Sobic.009G255000	9	62710547	62715408
Sobic.009G255100	9	62715394	62718684

Sobic.009G255200	9	62723941	62725335
Sobic.009G255300	9	62725251	62729180
Sobic.009G255400	9	62729402	62730686
Sobic.009G255500	9	62737342	62740507
Sobic.009G255600	9	62745183	62753917
Sobic.009G255650	9	62754017	62754559
Sobic.009G255700	9	62754086	62761724
Sobic.009G255800	9	62762496	62768663
Sobic.009G255900	9	62770415	62775783
Sobic.009G256000	9	62779356	62784672
Sobic.009G256100	9	62784579	62788568
Sobic.009G256200	9	62788578	62790499
Sobic.009G256300	9	62794094	62807271
Sobic.009G256400	9	62809003	62823521
Sobic.009G256500	9	62824557	62828944
Sobic.009G256700	9	62888450	62892806
Sobic.009G256800	9	62902344	62905140
Sobic.009G256900	9	62919352	62927162
Sobic.009G257100	9	62946578	62948996
Sobic.009G257200	9	62950057	62954708
Sobic.009G257300	9	62969808	62974860
Sobic.009G257400	9	62974740	62977432
Sobic.009G257500	9	62978820	62982745
Sobic.009G257600	9	62982901	62988044
Sobic.009G257700	9	62988101	62990744
Sobic.009G257800	9	62996289	63000944
Sobic.009G257900	9	63003343	63008851
Sobic.009G258000	9	63010382	63012309
Sobic.009G258100	9	63014606	63021943
Sobic.009G258150	9	63021944	63022881
Sobic.009G258300	9	63032905	63034903
Sobic.009G258400	9	63035171	63040041
Sobic.009G258500	9	63040077	63046389

Supplemental Table S4. Genes located within the plausible window, defined based on linage disequilibrium, to represent potential causal variations associated with the three most significant SNPs identified in the GWAS analysis for flowering time variation.
